# The quinone-based derivative, HMNQ induces apoptotic and autophagic cell death by modulating reactive oxygen species in cancer cells

**DOI:** 10.18632/oncotarget.21005

**Published:** 2017-09-18

**Authors:** Eun Byul Lee, Min Gyeong Cheon, Jun Cui, Yoo Jin Lee, Eun Kyoung Seo, Ho Hee Jang

**Affiliations:** ^1^ Department of Health Sciences and Technology, Graduate School of Medicine, Gachon University, Incheon, South Korea; ^2^ Department of Ophthalmology, Affiliated Hospital of Yanbian University, Yanji, Jilin, People’s Republic of China; ^3^ College of Pharmacy, Graduate School of Pharmaceutical Sciences, Ewha Womans University, Seoul, South Korea; ^4^ Department of Biochemistry, College of Medicine, Lee Gil Ya Cancer and Diabetes Institute, Gachon University, Incheon, South Korea

**Keywords:** anticancer drug, natural compound, reactive oxygen species (ROS), apoptosis, autophagy

## Abstract

8-Hydroxy-2-methoxy-1,4-naphthoquinone (HMNQ), a natural compound isolated from the bark of *Juglans sinensis* Dode, displays cytotoxic activity against various human cancer cells. However, the molecular mechanism of the anticancer effect is unclear. In this study, we examined the cytotoxic mechanism of HMNQ at the molecular level in human cancer cells. Cells were treated with HMNQ in a dose- or time-dependent manner. HMNQ treatment inhibited cell viability, colony formation and cell migration, indicating that HMNQ induced cancer cell death. HMNQ-treated cells resulted in apoptotic cell death through PARP-1 cleavage, Bax upregulation and Bcl-2 downregulation. HMNQ was also observed to induce autophagy by upregulating Beclin-1 and LC3. Furthermore, HMNQ induced reactive oxygen species (ROS) production, which was attenuated by the ROS scavengers, NAC and GSH. Finally, HMNQ increased expression of JNK phosphorylation and the JNK inhibitor SP600125 rescued HMNQ-induced cell death, suggesting that the cytotoxicity of HMNQ is mediated by the JNK signaling pathway. Taken together, our findings show that HMNQ exhibits anticancer activity through induction of ROS-mediated apoptosis and autophagy in human cancer cells. These data suggest the potential value of HMNQ as a natural anticancer drug.

## INTRODUCTION

Cancer is the second leading cause of death globally and one in six people dies of cancer [1]. Discovery and development of anticancer drugs remain a major focus of pharmaceutical companies, even though many anticancer drugs have been available. This is because anticancer drugs to be developed in the future must be less toxic, safe and effective for cancer treatment [2, 3]. Recently, interest in and expectations of natural anticancer compounds have been increasing [4–6].

Cancer cells rapidly grow and divide, and constantly produce new cells. To prevent cancer cell proliferation and metastasis, anticancer drugs should exhibit cytotoxicity that inhibits proliferative ability or induces cell death [7, 8]. Three major pathways induce cell death [9, 10]. Apoptosis, also called programmed cell death, can be divided into the extrinsic pathway (death receptor pathway) and intrinsic pathway (mitochondrial pathway). Extrinsic pathway is mediated by complexes formed through binding of ligands to death receptors. These complexes activate procaspase-8 and induce apoptosis by inducing caspase cascades [11, 12]. Intrinsic pathway is mediated by the Bcl-2 family, that controls mitochondrial permeability. Anti-apoptotic protein Bcl-2 inhibits cytochrome c release in the outer mitochondrial wall. But pro-apoptotic proteins Bax, Bad, and Bim translocate to mitochondria following stress signaling, and then cytochrome c is released by pro-apoptotic complexes. Released cytochrome c triggers the caspase-3/9 signaling cascade to promote apoptosis [13–15]. Second, autophagy, a genetically programmed and evolutionarily conserved catabolic process, selectively breaks down abnormal or damaged proteins and organelles. The autophagy process is initiated by vesicle nucleation formed through multiprotein complex, including Beclin-1, and forms a phagophore through membrane elongation of Atg12-Atg5. At the same time, soluble LC3 is cleaved by Atg4 to form LC3-1, that is again converted to LC3-II through conjugation to phycoerythrin (PE) by Atg7 and Atg3, and is attached to the autophagosome membrane. Attached LC3 and ubiquitin-binding protein, SQSTM1, isolates cargo molecules into the autophagosome, that fuse with lysosomes to form autolysosomes, that ultimately terminate the autophagy process by lysosomal hydrolysis [16–18].

Since autophagy is precisely regulated, autophagic cell death has recently been recognized as a subset of programmed cell death, and has attracted attention as a target for cancer treatment [19–22]. However, necrosis, one of the last cell death pathways, is controversial despite some form of programmed necrotic cell death (necroptosis) [23, 24]. These cell deaths are induced by a variety of genotoxic stresses, including UV and oxidative stress [25, 26]. It is also triggered by excessive reactive oxygen species (ROS) that directly impairs mitochondria, that play a critical role in cell survival through regulation of metabolism, energy homeostasis and respiration [27, 28]. Therefore, ROS is often used as a target of cancer treatment [29–31].

c-Jun amino-terminal kinases (JNKs) are members of mitogen-activated protein kinases (MAPKs), serine/threonine-specific protein kinases involved in diverse cellular functions including proliferation, stress response, cell survival and apoptosis [32, 33]. In apoptosis, JNKs are activated by a variety of environmental stresses including oxidative stress, and activated JNKs promote apoptosis by increasing expression of pro-apoptotic genes or modulating pro- and anti-apoptotic proteins through phosphorylation [34, 35]. Therefore, research on anticancer agents using ROS inducing compounds that cause JNK mediated apoptosis is expected [28]. ROS-inducing natural products promote apoptosis through activating JNK, p38 MAPK signaling pathways [36–38].

8-Hydroxy-2-methoxy-1,4-naphthoquinone (HMNQ) isolated from the bark of *Juglans sinensis* Dode, has potent cytotoxicity against human cancer cells [39]. However, the molecular mechanism of HMNQ-induced anticancer activity is unclear. In this study, we investigated molecular mechanism of HMNQ-induced apoptosis in MAPK signaling pathway and ROS production. We demonstrate that HMNQ exhibits anticancer activity through induction of ROS-mediated apoptosis by activation of the JNK pathway. This study reveals for the first time that HMNQ can also induce ROS-mediated autophagic cell death. Results suggest that HMNQ may be used as a potent natural anticancer drug.

## RESULTS

### HMNQ, a cytotoxic compound from *Juglans sinensis* Dode

**Figure 1:**
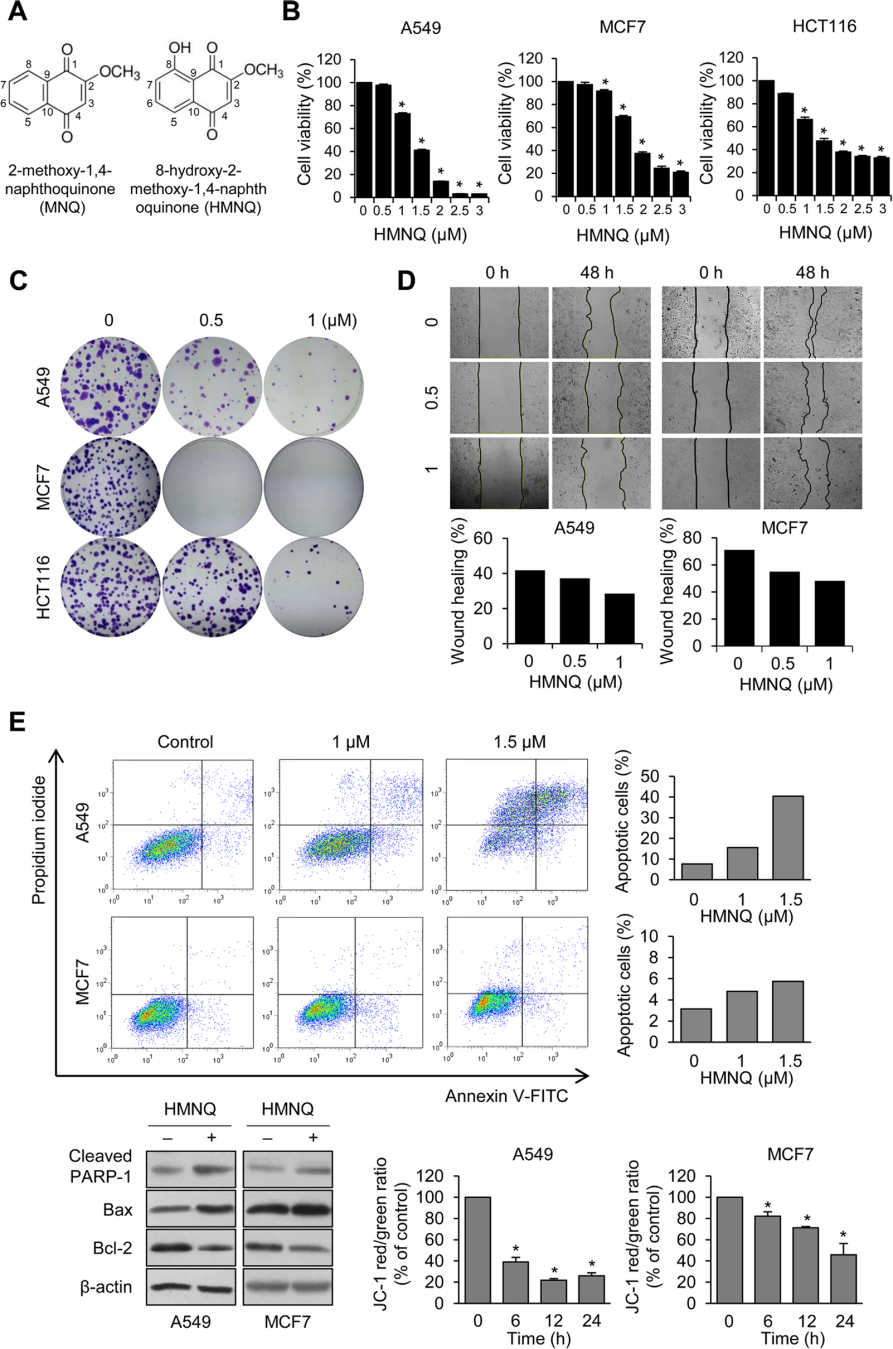
HMNQ inhibits cell proliferation by mitochondrial-mediated apoptosis (**A**) Chemical structures of 2-methoxy-1,4-naphthoquinone (MNQ) and 8-hydroxy-2-methoxy-1,4-naphtoquinone (HMNQ). (**B**) Cells were treated with the indicated dose of HMNQ for 24 h, and cell viability was measured. (**C**) Cells were treated with the indicated concentration of HMNQ for 2 weeks. Colonies were stained with 0.1% crystal violet. (**D**) Cells were scratched and treated with HMNQ for 48 h. Wound healing was quantified from the area of cell layer using Image J. (**E**) Cells were treated with HMNQ for 24 h and then stained with Annexin-V and propidium iodide (PI). Apoptotic cells were analyzed by flow cytometry. Levels of proteins were evaluated by western blot analysis after treatment with 1.5 μM HMNQ for 24 h. Mitochondrial membrane potential was monitored by JC-1 dye after incubation with 1.5 μM HMNQ for the indicated times. Plots are means ± SD, *n* = 3. ^*^P < 0.05 vs. control.

We previously reported that compounds from *Juglans sinensis* Dode have anti-proliferative activity [39]. Based on these results, we suggested that these compounds may be potential therapeutic agents for cancer treatment. To investigate the applicability of the compounds as practical anticancer drugs, we conducted the present follow-up study in various human cancer cell lines. Among 17 compounds isolated from *J. sinensis* Dode, compound 1 (Figure 1A, right) showed the strongest anti-proliferative effect. Compound 1 is a structure formed by a hydroxyl group inserted at carbon site eight of 2-methoxy-1,4-naphthoquinone (MNQ) (Figure 1A, left). Thus, Compound 1 was termed 8-hydroxy-2-methoxy-1,4-naphthoquinone (HMNQ).

### HMNQ inhibits growth and migration of cancer cells through mitochondrial-mediated apoptosis

Quinone-based derivatives such as HMNQ, showed cytotoxicity against several cancers [39], but it is unclear how the effects are mediated and expressed in cells. To confirm whether HMNQ has cytotoxicity toward cancer cells, we selected three cancer cell lines (A549 lung cancer, MCF7 breast cancer, HCT116 colon cancer cells) that had been previously shown to be potently affected by HMNQ and measured cell viability (Figure 1B). Cell viability was significantly decreased in a HMNQ dose-dependent manner, especially at concentrations above 1.5 μM. To examine the anticancer activity of HMNQ, colony forming and wound healing assay were performed (Figure 1C and 1D). As expected, HMNQ efficiently inhibited colony formation and cell migration at low concentrations compared to untreated cells. These data indicated that HMNQ induced cancer cell death. To investigate whether HMNQ inhibits the growth and migration of cancer cells by apoptosis-induced cell death, an Annexin V and PI apoptosis assay was performed in A549 and MCF7 cells treated with HMNQ for 24 h (Figure 1E, upper panel). HMNQ induced apoptosis in a dose-dependent manner, and the apoptotic proportion was increased up to about 40% in A549, but less than 10% in MCF7. It is also expected to increase the apoptosis rate at higher concentrations, not at low concentrations such as 1.5 μM, in MCF7 cells. The values shown in the graph included both early and late apoptosis. Furthermore, expression of apoptosis related proteins were assessed (Figure 1E, lower left quadrant). Cleavage of PARP-1, upregulation of pro-apoptotic protein, Bax and downregulation of the anti-apoptotic protein, Bcl-2, were observed in both A549 and MCF7 cells treated with HMNQ. Apoptosis is associated with dysfunction of the mitochondrial membrane potential (MMP) [13, 15, 40]. To investigate whether HMNQ causes dysfunction of MMP, we analyzed the integrity of mitochondrial functions after staining with JC-1 using multi-label counter followed by treatment with or without 1.5 μM HMNQ (Figure 1E, lower right quadrant). The ratio of JC-1 fluorescence intensity was significantly reduced from 100% to 25% in A549 cells, and from 100% to 45% in MCF7 cells by HMNQ treatment, revealing that HMNQ disrupts the MMP in A549 and MCF7 cells. These results suggest that the decrease of cell viability by HMNQ treatment is due to apoptosis through disruption of MMP. Overall, these results indicate that HMNQ inhibits the proliferation of cancer cells through mitochondrial mediated cell death.

### HMNQ induces ROS production, and is inhibited by ROS scavengers

**Figure 2:**
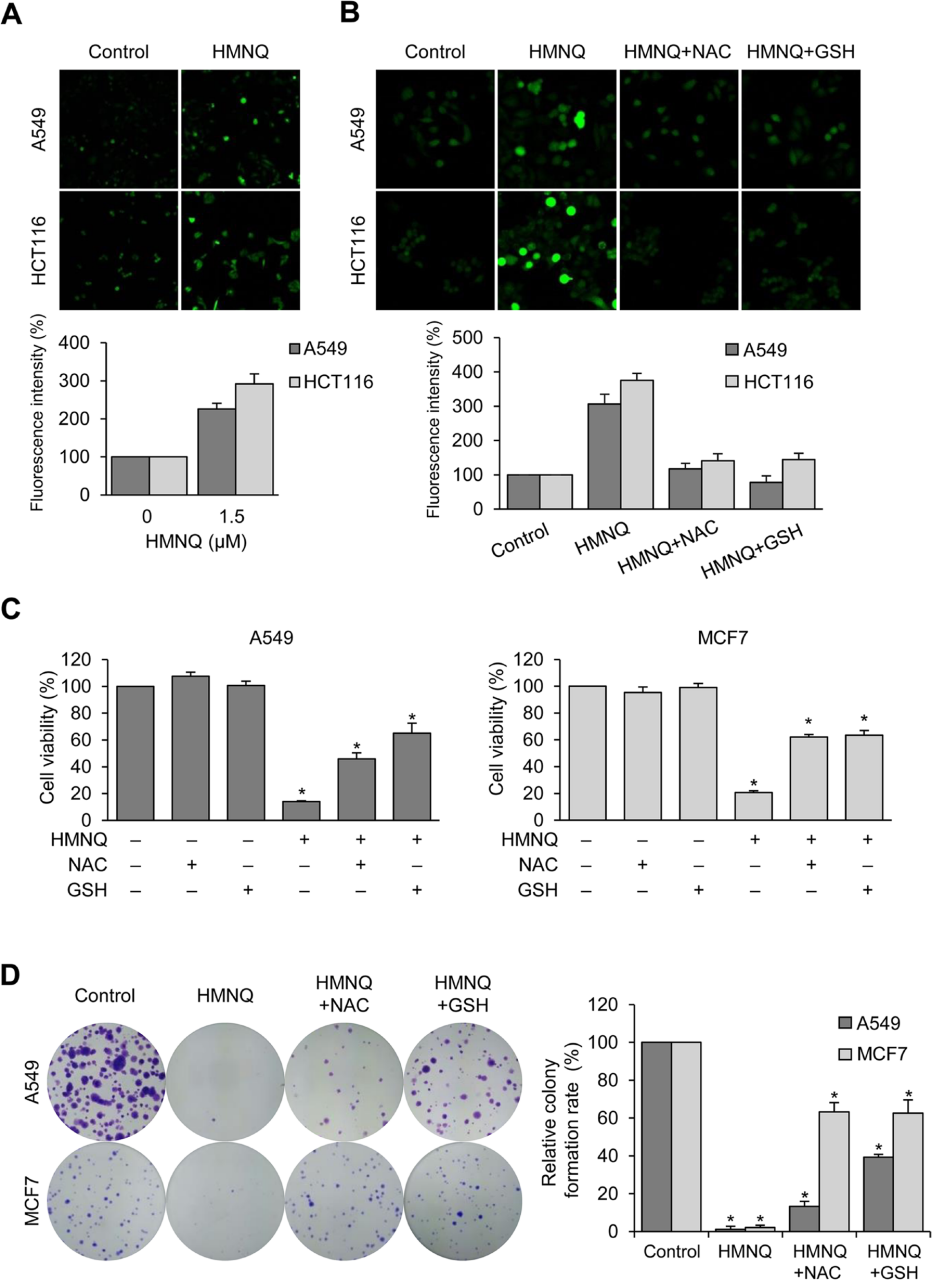
HMNQ inhibits cell proliferation through the generation of ROS (**A**) Cells were treated with 1.5 μM HMNQ for 12 h. Intracellular ROS levels were measured by DCF-DA staining, and the fluorescence images were obtained by confocal microscopy. (**B**) Cells were pretreated with ROS scavengers 5 mM NAC or 5 mM GSH for 1 h and then treated with 1.5 μM HMNQ for 12 h. The fluorescence intensity was assessed using Image J software. (**C**) Cells were treated with 1.5 μM HMNQ for 24 h in the absence or presence of 5 mM NAC and 5 mM GSH, and cell viability was measured. (**D**) Cells were treated with 1 μM HMNQ in the absence or presence of ROS scavengers (same as above) for two weeks. Colonies were photographed, and the number of colonies were counted using Image J. Plots are means ± SD, *n* = 3. ^*^P < 0.05 vs. control.

Excessive production of ROS can cause oxidative damage to intracellular organelles, including mitochondria, and it usually induces mitochondrial-mediated cell death [27, 28]. It has been also reported that increased ROS generation is related to destruction of MMP [27, 41]. To investigate whether HMNQ induces ROS production, the levels of intracellular ROS were analyzed by laser microscopy after exposure to HMNQ using DCF-DA staining (Figure 2A). When exposed to 1.5 μM HMNQ for 12 h, the ROS levels were increased approximately 2–3 fold compared to untreated controls in both cell lines. In Figure 2B, to confirm the intracellular ROS production by HMNQ, DCF-DA intensity was analyzed again after pretreatment with an ROS scavenger, NAC or GSH. NAC- or GSH-treated cells displayed inhibited ROS generation compared to cells treated with HMNQ alone, suggesting that HMNQ increases ROS levels, which can be prevented by NAC and GSH treatment. Next, the cell viability and colony forming assays were performed to assess whether cell viability could be restored by the NAC- or GSH-mediated reduction of ROS (Figure 2C and 2D). Cell viability and colony formation rates were significantly recovered by NAC or GSH treatments against HMNQ mediated cytotoxicity. These results indicate that HMNQ inhibits cell viability through ROS production, which can be rescued by the ROS scavengers, NAC and GSH.

### HMNQ induces apoptosis by phosphorylation of JNK

**Figure 3:**
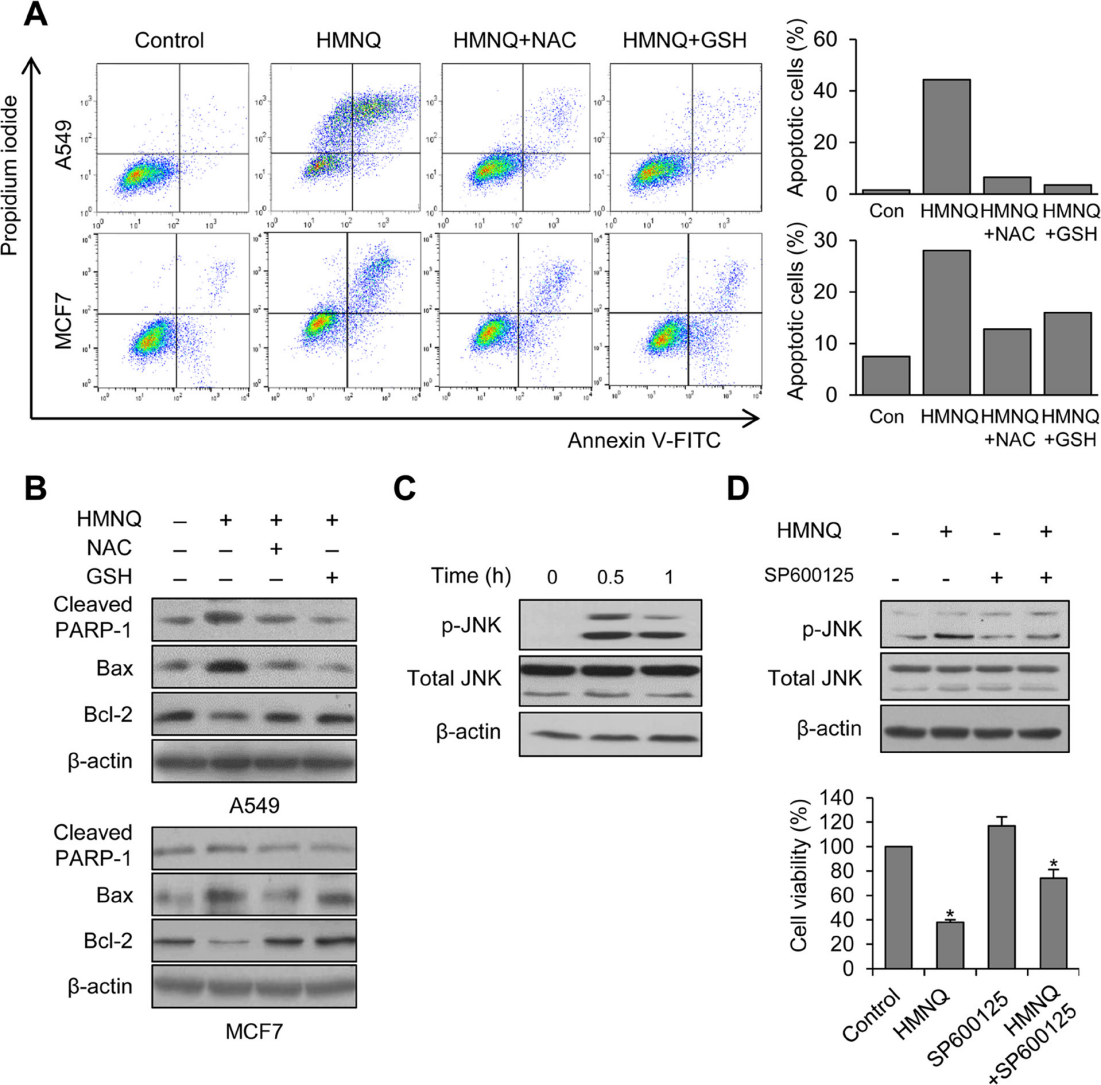
HMNQ-induced apoptosis through the JNK signaling pathway (**A**) Cells were pretreated with 5 mM NAC and 5 mM GSH for 1 h and then incubated with 1.5 μM HMNQ for 24 h. Flow cytometry was performed to measure apoptotic cells. (**B**) Cells were treated as described above. Levels of proteins were detected by western blot analysis. (**C**) MCF7 cells were treated with 1.5 μM HMNQ for the indicated times. JNK phosphorylation was detected by western blot analysis. (**D**) MCF7 cells were pretreated with JNK inhibitor, SP600125 (10 μM) for 1 h and then incubated with 1.5 μM HMNQ for 1 h. JNK phosphorylation was detected by western blot analysis. MCF7 cells were treated with 1.5 μM HMNQ for 24 h in the absence or presence of SP600125 (10 μM) and cell viability assay was performed. Plots are means ± SD, *n* = 3. ^*^P < 0.05 vs. control.

To examine whether ROS generation is associated with HMNQ-induced apoptosis, we further confirmed apoptosis in A549 and MCF7 cells by pre-incubation with NAC and GSH before HMNQ treatment using flow cytometry. Although there were differences in the amount of change between the two cell lines, NAC and GSH pretreatment efficiently reduced the population of apoptotic cells compared to cells treated with HMNQ alone (Figure 3A). In addition, western blotting analysis also showed that significantly decrease of cleaved PARP-1, Bax, and increased Bcl-2 by NAC and GSH pretreatment (Figure 3B). These results suggest that the ROS scavengers rescued cells from HMNQ-induced cytotoxicity. The JNK pathway is an important MAPK signaling pathway for apoptosis through ROS generation [34, 35]. To investigate whether activation of JNK pathway is involved in HMNQ-induced apoptosis, MCF7 cells were treated with HMNQ (Figure 3C). When 1.5 μM HMNQ was treated for 0.5 and 1 h, the levels of phosphorylated JNK, the active form, significantly increased with time. Compared with the increase of JNK phosphorylation, there were no changes in total JNK levels. Thus, the apoptosis was mediated through an increase in activity of JNK rather than upregulation of expression. Furthermore, the cells were pretreated with JNK inhibitor, SP600125, and then incubated with HMNQ for 1 h (Figure 3D, upper panel). As expected, western blotting showed that pretreatment with SP600125 efficiently reverse HMNQ-induced JNK phosphorylation in MCF7 cells. It was confirmed that the same pattern was obtained from the cell viability (Figure 3D, lower panel). Overall, these results suggest that HMNQ induces apoptosis through the JNK signaling pathway by decreasing MMP via ROS production.

### HMNQ induces autophagy-mediated cell death through ROS generation

**Figure 4:**
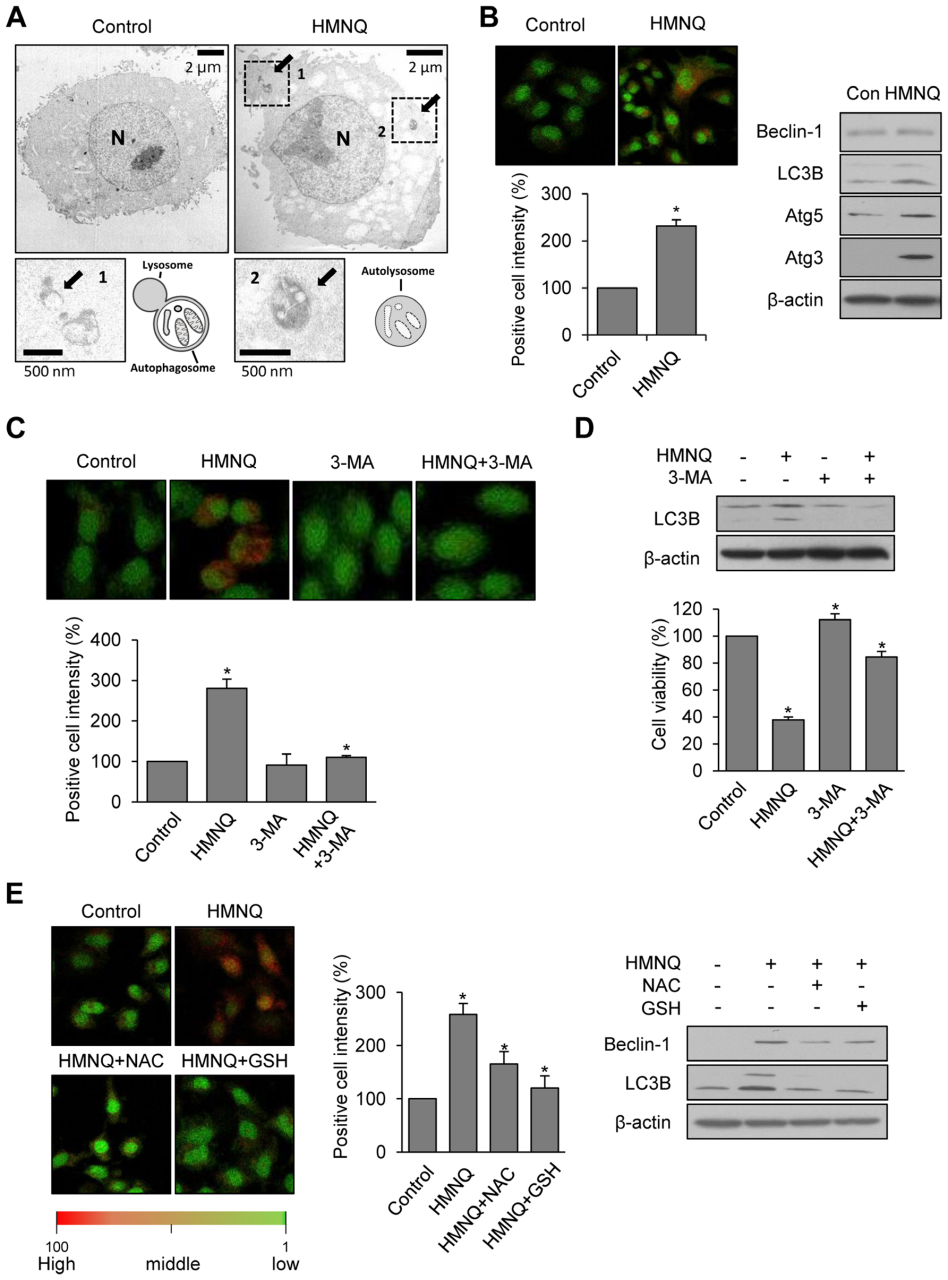
HMNQ induces autophagy-mediated cell death through ROS generation (**A**) A549 cells were treated with or without 1 μM HMNQ for 24 h and examined by TEM. (**B**) A549 cells were treated with 1.5 μM HMNQ for 24 h and stained with acridine orange dye for 30 min. Autophagic cells were visualized as orange by fluorescence microscopy and intensity was measured using Image J software. Western blotting was performed to detect Beclin-1, LC3B, Atg3 and 5. (**C**) After pretreatment of 3-MA for 1 h, the A549 cells were incubated with 1.5 μM HMNQ for 24 h and stained with acridine orange. The fluorescence was observed under confocal microscope and intensity was measured using Image J. (**D**) The levels of LC3B were detected by western blotting and cell viability was measured. (**E**) A549 cells were treated with 1.5 μM HMNQ containing with 5 mM NAC or 5 mM GSH for 24 h. Acridine orange dye was used to stain late autophagic vacuoles. The levels of Beclin-1, LC3B were detected by western blotting. Plots are means ± SD, *n* = 3. ^*^P < 0.05 vs. control.

Autophagy, an evolutionarily conserved catabolic process, is critical for cell fate, such as survival and death. Autophagy and apoptosis are considered forms of programmed cell death. [18–20]. For these reasons, autophagy has become the target of anticancer drugs [22, 42]. Therefore, we examined whether HMNQ could induces autophagy-mediated cell death. To determine whether autophagy was occurred by treatment of HMNQ, cells were observed with TEM (Figure 4A). Compared with the control, autophagic behavior was observed in cells treated with 1 μM HMNQ for 24 h. The photographs were taken at high resolution, and each arrow in the picture denotes the autophagolysosome (fusion of the autophagosome with the lysosome) and autolysosome. The smaller boxes below are 4x magnification photographs, and cartoons corresponding to autophagy process in each photographs. To confirm that autophagy is induced by HMNQ, the phenomenon was observed using acridine orange dye, which can stain autophagic cells under the same conditions (Figure 4B, left). Autophagic cells were visualized in orange by fluorescence microscopy in HMNQ-treated cells. It was also confirmed that autophagy-related key molecules Beclin-1, LC3B, Atg3 and Atg5 were increased in HMNQ-treated cells using western blotting (Figure 4B, right). After pretreatment of the autophagy inhibitor, we investigated whether HMNQ-induced autophagy could be blocked by the autophagy inhibitor 3-MA, using measurement the intensity of acridine orange (Figure 4C), and then checked cell viability and related molecules (Figure 4D). Cell viability was restored at a similar rate to the decrease in acridine orange fluorescence intensity. Next, we investigated whether autophagy can be induced by ROS generation and restored by the ROS scavengers, NAC and GSH. Similar results were obtained with apoptosis through staining of acridine orange dye and western blotting (Figure 4E). These results suggest that HMNQ can also induce autophagic cell death.

Altogether, these results indicate that HMNQ inhibits cancer cell proliferation and viability by inducing ROS-dependent apoptosis and autophagy. Our results indicate the potential value of HMNQ as a natural anticancer drug.

## DISCUSSION

Drug development using natural derivatives has progressed steadily as a treatment for various human diseases including cancer, due to the low toxicity and excellent efficacy of the discovered derivatives [43, 44]. Quinone or quinone-derived compounds have anticancer activity against several human cancer cells [45, 46]. For example, MNQ showed cytotoxic effects such as ROS-mediated apoptosis, in A549 cells [37] and inhibition of cell migration and invasion in MDA-MB-231 cells [47]. In addition, our previous study first showed the anti-proliferative activity in HMNQ isolated from *J. sinensis* Dode [39]. But, its molecular mechanism of action has been unknown.

**Figure 5:**
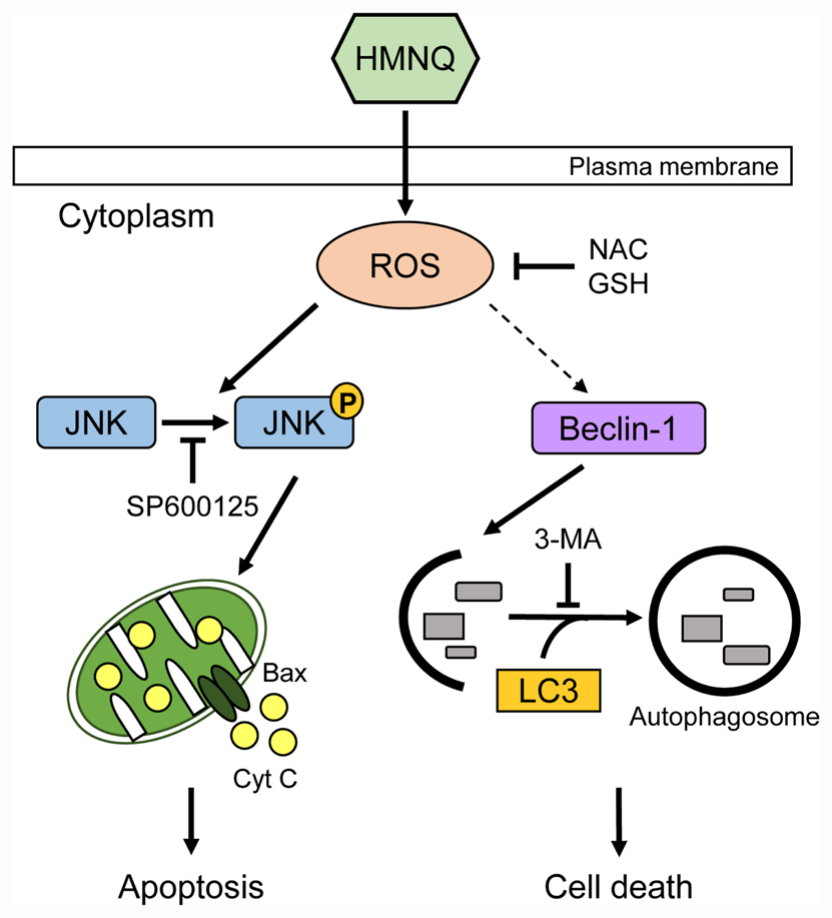
Schematic representation of HMNQ-induced apoptosis and autophagy HMNQ treatment induces the generation of ROS. Phosphorylated JNK by ROS activates pro-apoptotic protein, Bax, thus facilitating the release of Cyt C outside the mitochondria, thereby causing downstream cascades that leads to DNA fragmentation. Finally, apoptotic cell death is induced. Autophagy is also mediated by HMNQ-induced ROS, which leads to cell death by activation of Beclin-1 and LC3.

Compounds derived from quinone elicit production of ROS [48, 49]. In addition, several previous studies have shown that high levels of ROS induce oxidative damage and activate apoptotic pathway, and ultimately leading to cell death [35]. We hypothesized that HMNQ increases intracellular ROS and induces apoptotic cell death. Presently, we demonstrate that HMNQ induces apoptosis of cancer cells through an ROS-dependent JNK signaling pathway (Figures 3 and 5). We detected ROS generation and an intrinsic pathway for the induction of apoptosis by HMNQ treatment in human cancer cells. These findings were confirmed through HMNQ-induced ROS generation (Figure 2A), MMP disruption (Figure 1E lower right quadrant) and expression of apoptosis-associated proteins (Figure 1E, lower left quadrant). In addition, HMNQ-induced apoptosis was caused by ROS generation, since the ROS scavengers, NAC and GSH, suppressed both HMNQ-induced ROS production (Figure 2B) and apoptosis (Figure 3A and 3B). Over-production of intracellular ROS triggers the MAPK signaling pathway [28], which is involved in the regulation of many cellular processes including cell proliferation, differentiation, development, inflammation and apoptosis. ERK, JNK and p38 kinases are key members of the MAPK family involved in stress-induced signaling pathway [50]. Presently, HMNQ activated the JNK pathway (Figure 3C) as confirmed by the JNK inhibitor, SP600125 (Figure 3D). Inhibition of JNK also reduced HMNQ-induced cell death (Figure 3D, lower panel), indicating that the HMNQ-induced oxidative stress stimulates activation of JNK pathway leading to the intrinsic apoptosis pathway. Similarly, MNQ and panaxydol have also been shown to induce cancer cell apoptosis through the JNK pathway [37, 51]. Thus, the JNK pathway is involved in HMNQ-induced apoptosis in human cancer cells.

Autophagy and apoptosis are major pathways that determine the cell’s fate. They have been regarded as a tool for programmed cell death, and many of today’s anticancer agents aim at apoptosis as well as autophagy control [9]. Although quinone-related autophagy has not been reported as much as apoptosis, there are some papers that quinone derivatives induce ROS-dependent autophagy [52, 53]. We therefore investigated whether HMNQ could also induce autophagy (Figure 4). We found that autophagy was induced by ROS-dependent cell death in HMNQ treated cancer cells (Figures 4E and 5). In addition, autophagy was observed by TEM and acridine orange dye after HMNQ treatment for 24 h (Figure 4A and 4B). Reduction of autophagy positive cells and related molecules were observed following the use of the autophagy inhibitor, 3-MA (Figure 4C and 4D). Unfortunately, unlike the apoptosis study, the details of the autophagy mechanism were not revealed. However, based on the previous reports, we assume that ROS-dependent autophagy is regulated via the phosphoinositol-3-kinse/AKT or nuclear factor-kappa B pathway. This will be investigated [54, 55].

In conclusion, HMNQ inhibits cell viability through ROS-dependent apoptosis and autophagy in several cancer cell lines. Specifically, the increase of ROS by HMNQ treatment induces phosphorylation of JNK, leading to mitochondrial mediated apoptotic cell death.

## MATERIALS AND METHODS

### Cell culture

A549 human lung cancer cells and HCT116 human colon cancer cells were cultured in RPMI 1640 supplemented with 10% fetal bovine serum (FBS; Capricorn Scientific, Ebsdorfergrund, Germany) and 100 U/ml Penicillin/Streptomycin (Capricorn Scientific) at 37°C in an atmosphere of 5% CO_2_. MCF7 human breast cancer cells were maintained in Dulbecco’s modified Eagle’s medium (DMEM).

### Cell viability assay

Cell viability was analyzed according to the manufacturer’s instructions using the EZ-CyTox Cell Viability Assay Kit (EZ-CYTOX; Daeillab Service, Seoul, Korea) [56]. A549, MCF7 and HCT116 cells (5 × 10^3^ cells/well) were seeded on a 96-well plates, and then treated with various concentration of HMNQ for 24 h. EZ-Cytox solution was then added to each well, and incubated at 37°C for 4 h. The absorbance was measured at 450 nm using a VERSA max microplate reader (Molecular Devices, Silicon Valley, CA, USA) and the percentage of viable cells compared to the untreated cells was calculated. The results were explored as cell viability (%) = (mean absorbency in test wells/mean absorbance in control wells) × 100.

### Colony formation assay

A549, MCF7 and HCT116 cells (0.5 × 10^3^ cells/well) were seeded on 6-well plates, and then treated with HMNQ. The plates were incubated at 37 °C in a 5% CO_2_ incubator. After 2 weeks, the cells were washed with PBS, fixed with 4% formaldehyde for 30 min, and stained with 0.1% crystal violet (Sigma-Aldrich, St. Louis, MO, USA). The colonies were photographed and counted on triplicate dishes, and independent experiments were repeatedly done. Relative number of colonies = (number of HMNQ treated cells /number of control cells) × 100 %.

### Wound healing assay

A549 and MCF7 cells were seeded on 6-well plates, and cultured until cells were confluent. After that, cells were scratched and treated with or without HMNQ for 48 h. The cell morphology was observed under inverted phase-contrast microscopy. Three fields in each well were photographed and the number of migrated cells counted.

### Annexin V/propidium iodide (PI) staining

Apoptosis analysis was performed according to the manufacturer’s instructions using EzWay™ Annexin V-FITC apoptosis detection kit (KOMA Biotech, Seoul, Korea) [57]. A549 and MCF7 cells were pretreated with or without N-acetylcysteine (NAC; Sigma-Aldrich) and glutathione (GSH; Sigma-Aldrich) for 1 h, and then treated with various concentrations of HMNQ. After 24 h incubation, the cells were washed with PBS and stained with Annexin V-FITC and PI for 15 min at room temperature. Apoptotic cells were analyzed using flow cytometer (Becton Dickinson, San Jose, CA, USA).

### Western blotting analysis

HMNQ-treated A549 and MCF cells were lysed using cell lysis buffer (150 mM NaCl, 20 mM HEPES pH 7.5, 1% Triton X-100, 10% glycerol) with protease inhibitor cocktail (GenDEPOT, Austin, TX, USA) on ice for 10 min. After centrifugation at 12,000 × g for 15 min, the supernatant was used as a crude cell extract for sodium dodecyl sulfate-polyacrylamide gel electrophoresis. The resolved proteins were transferred to a nitrocellulose membrane. The antibodies used for Western blotting were: poly (ADP-ribose) polymerase-1 (PARP-1; (Santa Cruz Biotechnology, Dallas, TX, USA), Bax (Santa Cruz Biotechnology), Bcl-2 (Santa Cruz Biotechnology), JNK (Cell Signaling Technologies, Waltham, MA, USA), phospho-JNK (pJNK; Cell Signaling Technologies), LC3B (Cell Signaling Technologies), Beclin-1 (Cell Signaling Technologies) and β-actin (Cell Signaling Technologies).

### Measurement of mitochondria membrane potential (MMP)

MMP was monitored using JC-1 (Biotium, Fremont, CA, USA) [58]. A549 and MCF7 cells were seeded on 96-well plates and incubated with 1.5 μM HMNQ in a time-dependent manner. The cells were washed twice with cold PBS and stained with JC-1 for 15 min at 37°C in the dark. Fluorescence intensity was measured using a Wallac Victor3 1420 multi-label counter (Perkin-Elmer, Wellesley, MA, USA). The green and red JC-1 signals were measured at excitation and emission wavelengths of 485 and 535 nm, and 535 and 590 nm, respectively.

### ROS analysis

Intracellular ROS levels were measured using 2′,7′-dichlorofluorescin diacetate (DCF-DA; Invitrogen, Carlsbad, CA, USA) according to the manufacturer’s instructions. Briefly, cells were seeded on 12-well plates and then pretreated for 1 h with or without 5 mM NAC and 5 mM GSH (Sigma-Aldrich). After co-treatment with 1.5 μM HMNQ for 12 h, the cells were incubated with fresh complete medium containing 20 μM DCF-DA for 15 min at 37°C in the dark. The DCF fluorescence intensity was measured using a fluorescence microscope. Images were captured using an Axio Imager Z1 (Zeiss, Jena, Germany).

### Acridine orange staining

A549 cells were seeded on 24-well plates and treated with HMNQ for 12 h. The cells were rinsed cells twice with cold PBS and incubated with 5 μM acridine orange solution (Invitrogen, Carlsbad, CA, USA) [59] in the dark. Cell images were obtained using the aforementioned Axio Imager Z1 microscope. The intensity of fluorescence was measured with Image J software (NIH, Bethesda, MD, USA). Fluorescence intensity of positive cells (%) = (Intensity of HMNQ treated cells/intensity of control cells) * 100.

### Transmission electron microscopy (TEM)

A549 cells were seeded on 100 mm plates. HMNQ was treated for 24 h and then harvest in phosphate buffer. After centrifugation at 3000 rpm, the cells were washed twice with 0.1 M phosphate buffer, fixed with 4% formaldehyde at 4°C for 2 h, washed twice and then dehydrated to graded ethanol series (50%, 70%, 95% and 100%) for 15 min at each concentration.

### Statistical analysis

All results are expressed as mean ± standard error (SEM). Statistical comparisons were determined using two-tailed unpaired Student’s *t*-test. Multiple comparisons were analyzed using one-way analysis of variance followed by a *post hoc* test (Turkey’s multiple comparison test). *P* < 0.05 was considered statistically significant.
